# Income-related disparities in the value of health care in South Korea

**DOI:** 10.1093/haschl/qxaf145

**Published:** 2025-07-22

**Authors:** Sungchul Park, Karen Eggleston, Young Kyung Do, David M Cutler

**Affiliations:** Department of Health Policy and Management, College of Health Science, Korea University, 145 Anam-ro, Seongbuk-gu, Seoul 02841, Republic of Korea; Shorenstein Asia-Pacific Research Center, Stanford University, 616 Jane Stanford Way Stanford University Stanford, Stanford, CA 94305, United States; National Bureau of Economic Research, Cambridge, MA 02138, United States; Department of Health Policy and Management, College of Medicine, Seoul National University, 103 Daehak-ro, Jongno-gu, Seoul 03080, Republic of Korea; Institute of Health Policy and Management, Seoul National University Medical Research Center, 103 Daehak-ro, Jongno-gu, Seoul 03080, Republic of Korea; National Bureau of Economic Research, Cambridge, MA 02138, United States; Department of Economics, Harvard University, 1875 Cambridge Street, Cambridge, MA 02138, United States; Kennedy School of Government, Harvard University, 1875 Cambridge Street, Cambridge, MA 02138, United States; T. H. Chan School of Public Health, Harvard University, 1875 Cambridge Street, Boston, MA 02115, United States

**Keywords:** value of health care, health care spending, quality-adjusted life expectancy, health disparity, income, South Korea

## Abstract

**Introduction:**

Health care spending in South Korea is associated with improvements in health. However, it remains unclear whether the value of this spending is equally distributed across income groups.

**Methods:**

We analyzed lifetime health care spending and quality-adjusted life expectancy (QALE) by income quintile among South Korean adults from 2010 to 2018. We then calculated the ratio of changes in health care spending to changes in QALE to estimate the value of health care spending across income groups. Additionally, we investigated mechanisms underlying income-related differences in the value of health care.

**Results:**

Assuming 80% of QALE gains are attributable to health care, adults in the lowest-income quintile received the least value, incurring $78 209 per QALE gained. However, middle- and higher-income quintiles achieved greater value ($47 831, $46 905, $31 757, and $53 889 from the second to highest quintile), although the highest value did not occur in the highest-income quintile. The higher spending per QALE gained in the lowest-income quintile reflects smaller improvements in QALE, likely driven by poorer baseline health and greater unmet needs.

**Conclusion:**

These findings highlight structural inequities in the South Korean health system and emphasize the need for targeted policies to promote equitable health care value.

Improving the value of health care has become a key policy priority for health systems worldwide.^1,2^ As health care spending continues to rise, many health systems have implemented value-based care reforms to align financial incentives with high-quality, cost-effective care.^1,2^ Concurrently, there is growing interest in empirically measuring the value of health care.^3-7^

This question is particularly relevant in South Korea, where health care spending has increased sharply over the past few decades.^8^ Between 2010 and 2019, real per capita health care spending increased from $1211 to $1903, growing nearly 6% annually.^8,9^ During this period, life expectancy increased from 80.5 to 83.7 years,^10^ and disability-adjusted life years (DALYs) per capita declined substantially.^9,11^ Evidence suggests that South Korea spent $12 924 per DALY averted during this period,^9^ comparable to or lower than estimates from other high-income countries.^3-5,12-16^ This indicates that, on average, health care spending in South Korea delivers reasonable value in improving health outcomes.

However, these aggregate trends may obscure important distributional concerns. Although the National Health Insurance system provides universal coverage, structural factors continue to drive disparities in health care use and outcomes. Income is a key contributor. In 2021, South Korea showed substantial income inequality, with the top 1% earning 11.7% of total income and the top 10% accounting for 34.4%.^17^ The country also has the highest old-age poverty rate in the Organisation for Economic Co-operation and Development (OECD), with over 40% of adults aged 65 and older living in relative poverty.^18^ Moreover, South Korea has the highest share of out-of-pocket spending relative to household medical costs among OECD countries,^19^ suggesting that financial barriers remain significant. These patterns raise critical policy questions about whether increases in health care spending are benefiting all segments of society equally.

Prior research documented differences in health care spending by income,^9^ but less is known about whether these spending patterns translate into comparable health outcomes across income groups. Between 2010 and 2019, spending grew most rapidly among higher-income groups, with the top quintile experiencing the largest increases.^9^ Notably, the lowest-income quintile, despite starting with the lowest spending in 2010, also experienced substantial increases by 2019. However, over this period, income-based differences in life expectancy continued to widen.^20^ These findings suggest that the health gains from increased spending have not been evenly shared across income groups, highlighting the need for policies that ensure more balanced improvements in population health.

To address this gap, we conducted two analyses. First, we examined trends in health care spending and health outcomes across income quintiles among South Korean adults between 2010 and 2018 and quantified the value of health care spending within each group. Second, we explored potential mechanisms underlying income-based differences in the value of health care.

## Methods

### Data

We conducted a cross-sectional study using multiple nationally representative data sources, including the Korean Health Panel Study (KHPS), the Korean National Health Insurance Service (NHIS), and the Korean National Health and Nutrition Examination Survey (KNHANES) for 2011-2018. The KHPS served as the primary data source, offering detailed information on household income, health care spending, and health-related quality of life (HRQoL). As a nationally representative annual panel survey, the KHPS employs a stratified two-stage cluster sampling design.

### Sample

We sampled adults aged 18 years and older. We excluded those in the bottom 10% of the household income distribution. This group was excluded because their low income may reflect poor health, and their demographic and socioeconomic characteristics often differ substantially from the rest of the population, complicating direct comparisons of the value of health care spending. The remaining sample was categorized into income quintiles each year based on annual household income, including both labor and asset income. We used this approach because existing evidence indicates that intragenerational income mobility in South Korea is relatively low, with income levels especially stable among the lowest and highest groups.^21,22^

### Outcomes

We examined two primary outcomes. Health care spending was measured as total medical expenditures, including costs for inpatient services, outpatient services, emergency services, and prescription medications. This comprised both NHIS payments and out-of-pocket expenses for services covered and not covered by the NHIS. Although NHIS data can track health care spending, these records do not provide exact income information and exclude costs for uncovered services. In contrast, the KHPS includes detailed income data and comprehensive spending information. The KHPS collects expenditure data through household interviews, where enumerators verify reported costs using receipts, medical bills, and credit card statements. This method ensures that spending estimates are based on documented evidence rather than solely on respondent recall. All spending measures were adjusted for inflation using the gross domestic product (GDP) deflator and are reported in 2021 US dollars.

Health outcomes were assessed using three indicators: HRQoL, life expectancy, and quality-adjusted life expectancy (QALE). HRQoL was measured using the visual analog scale (VAS), the EQ-5D instrument, and self-reported health status. The VAS asked respondents to rate their overall health on a continuous scale from 1 to 100 (Appendix),^23^ while the EQ-5D assessed five dimensions—mobility, self-care, usual activities, pain/discomfort, and anxiety/depression—which were weighted to produce a summary score.^24,25^ Both the VAS and EQ-5D scores were standardized on a 0-100 scale, with higher scores indicating better health. Self-reported health was originally measured on a five-point Likert scale and subsequently dichotomized into “good” (good, very good, or excellent) and “poor” (fair or poor) health categories.^26^

Life expectancy estimates were calculated using life table methods based on beneficiary enrollment data from the NHIS. As insurance premiums in South Korea are determined by annual household income, premium levels were used as a proxy for income. After excluding individuals in the lowest decile of the income distribution, the remaining population was stratified into income quintiles. Given the focus on the adult population, life expectancy at age 25 was estimated for each income group in each year. QALE was calculated by multiplying life expectancy at age 25 by HRQoL (VAS) scores, yielding a summary measure that reflects both longevity and the quality of life during those years (Appendix).

Furthermore, we analyzed secondary outcomes using data from the KHPS and the KNHANES. First, we assessed perceptions of the health care system based on each respondent's top concern, selected from seven mutually exclusive categories: high out-of-pocket costs, long wait times, low quality of care, authoritarian provider behavior, rising national expenditures, inequity in access, and other issues. Second, we constructed binary indicators across four domains. The first domain—health status—included comorbidities, functional limitations, and clinical indicators. The second domain—behavioral risk factors—included current smoking, obesity, and excessive alcohol consumption. The third—unmet health care needs—captured both financial and non-financial barriers to care. The fourth—utilization of preventive services—included general medical and dental checkups as well as cancer screenings.

### Statistical analysis

We analyzed baseline sample characteristics across income groups. Next, we examined trends in health care spending and health outcomes by income level from 2010 to 2018. Lower-income groups may have a higher proportion of older adults, which could confound the relationship between income and outcomes. To address these demographic differences, we used regression models that adjusted for age, sex, and their interaction. In accordance with the Institute of Medicine framework, which defines health care disparities as differences in care quality not explained by health care needs or preferences, we limited adjustments to demographic variables to avoid underestimating structural inequities.^27^ Using marginal effects from regression analyses, we estimated mean adjusted values for each group and year while holding all other variables constant, except the variable of interest. A two-part model was used for health care spending. Continuous health outcomes were analyzed using linear regression, while binary health outcomes were assessed using logistic regression.

To quantify the value of health care across income groups, we followed a three-step approach, building on prior research.^3,4^ First, we estimated adjusted annual outcomes for each income group from 2011 to 2018. We then projected lifetime health care spending by accounting for life expectancy and applying a 3% discount rate, ensuring spending estimates were aligned with the expected lifespan of each group. Second, we calculated the ratio of changes in projected lifetime health care spending to changes in QALE. Finally, we applied alternative assumptions regarding the attribution of health gains to health care. In the base case, we assumed that 100% of observed health improvements resulted from health care services. We also tested alternative assumptions based on previous literature: 50%, reflecting estimates that approximately half of life expectancy gains are due to medical advances,^28-30^ and 80%, reflecting that 80% of mortality decline in South Korea was due to causes potentially attributable to health care.^31^

To explore the mechanisms underlying income-based inequities in the value of health care, we conducted two types of secondary analyses. First, we assessed the distribution of self-reported concerns about the health care system. Second, we evaluated four domains to identify where income-based inequities in the value of care may arise. For these analyses, we conducted logistic regression after adjusting for age, sex, their interaction terms, and year and estimated demographic-adjusted differences by income.

For all analyses, survey weights were applied to ensure representativeness of the South Korean population.

## Limitations

First, our sample was limited to the non-institutionalized population, and the findings may not generalize to individuals in institutional settings. Second, both health care spending and health status were based on self-reported data, which may be subject to recall bias and reporting errors. Third, our measure of health care spending primarily captured direct medical costs and did not include other important components, potentially underestimating total health-related resources. Fourth, we assumed a uniform contribution of health care spending to health improvements across income groups. However, this effect may vary by income. Given data limitations, we examined this assumption by education level—which is highly correlated with income—and found that the contribution was relatively consistent across education groups. Fifth, our lifetime QALE estimates were conditional on survival to age 25, assuming individuals remain in the same income group over time. This approach may either under- or overestimate income-related differences, since those growing up in lower-income groups are less likely to survive to age 25, and older low-income individuals may have previously belonged to higher-income groups. Sixth, excluding the lowest 10% income group may overstate the value of health care, as this group is more likely to have severe disabilities and complex conditions. Finally, the findings are associational, not based on exogenous variation in income.

## Results

### Baseline characteristics

The final study sample included 64 382 person-year observations, with 12 879, 12 899, 12 856, 12 872, and 12 876 observations in the lowest through highest-income quintiles. Annual household income was right-skewed, with higher incomes concentrated in the upper quintiles (Appendix Figure A). In 2011, mean annual household income by quintile was $17,835, $30,443, $42,839, $57,655, and $93 185 (Appendix Table A) and remained relatively stable over the study period (Appendix Figure B).

Baseline characteristics revealed substantial demographic, socioeconomic, and health-related differences across income groups (Appendix Table A). Adults in lower-income quintiles were more likely to be older, have disabilities, receive medical aid, and experience a higher prevalence of comorbid conditions compared to those in higher-income quintiles. These disparities were particularly pronounced in the lowest-income quintile.

### Changes in health care spending and outcomes

**Figure 1.**
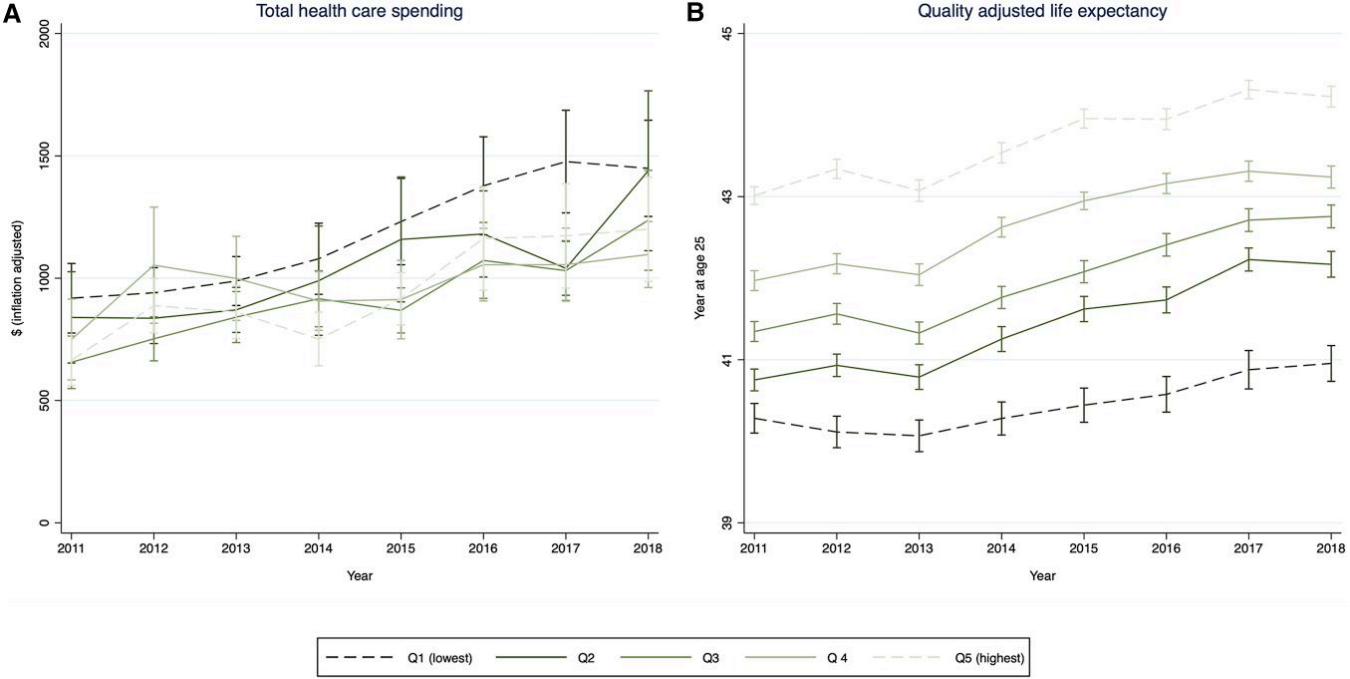
Trends in total health care spending and quality-adjusted life expectancy among adults by household income in South Korea. (A) Total health care spending. (B) Quality adjusted life expectancy. Sources: Authors' analysis of data from the Korean Health Panel Study and the Korean National Health Insurance Service for the years 2011-2018. Note: Total health care spending was defined as expenditures on medical care services, including inpatient, outpatient, and emergency room visits. This spending included payments made by the NHIS, patient cost-sharing, and expenses for non-covered services. All spending measures were inflation-adjusted to 2021 US dollars using the GDP deflator in Korea. Quality-adjusted life expectancy (QALE) was calculated by multiplying life expectancy by the visual analog scale (VAS) scores. The VAS scores were derived from respondents' self-rated overall health on a continuous scale. For more detailed calculations of VAS and QALE, see the Appendix. To compare health care spending and QALE across income groups and over time, regression models were employed. Key independent variables included household income quartiles, year, and their interaction. Covariates were age, sex, and the interaction between age and sex. A two-part model was used for health care spending and a linear regression model was used for QALE. To produce interpretable and comparable results, we calculated mean adjusted values for each income and year group. Survey weights were applied to ensure the sample characteristics were representative of the South Korean population.

Our adjusted analysis showed that from 2011 to 2018, both health care spending and QALE increased across all income groups in South Korea (Figure 1). Although baseline health care spending was highest in the lowest-income quintile, this group experienced the smallest relative increase over time. Specifically, spending increased annually by 6.6% in the lowest quintile ($918-$1448), 7.9% in the second ($839-$1439), 9.4% in the third ($656-$1236), 5.5% in the fourth ($749-$1096), and 8.8% in the highest quintile ($654-$1199). However, these differences were not statistically significant, as indicated by overlapping confidence intervals.

A similar pattern was observed in QALE. The lowest-income quintile had the lowest baseline QALE and experienced the smallest relative improvement over time—an increase of 0.7 years (1.7%) from 40.3 to 41.0. In comparison, QALE increased by 1.4 years (3.5%) in the second, 1.4 years (3.4%) in the third, 1.3 years (3.0%) in the fourth, and 1.2 years (2.8%) in the highest quintile. This disparity was primarily driven by differences in VAS scores rather than life expectancy (Appendix Figure C-D). While life expectancy increased across all income groups, it remained consistently lowest in the lowest-income quintile and declined over time. This trend was consistently observed across all three HRQoL measures.

### Value of health care

We observed clear income-based disparities in the value of health care among South Korean adults (Table 1). Adults in the lowest-income quintile derived the least value from health care spending. Under the assumption that 80% of QALE improvements were attributable to health care, the estimated cost per QALE gained in the lowest-income quintile was $78 209. In contrast, adults in the second through highest-income quintiles achieved greater value estimates of $47,831, $46,905, $31,757, and $53 889. Notably, the highest-income quintile did not yield the greatest value. Under a more conservative assumption—attributing only 50% of QALE gains to health care—the cost per QALE gained increased to $124 534 in the lowest quintile and to $76,529, $75,352, $51,180, and $86 676 in the remaining quintiles.

**Table 1.** Value of health care among adults by household income in South Korea.

	Average per capita spending, $		QALE: years of quality-adjusted life (remaining at age 25)		Value of medical care, $/QALE		
Household income	2011	2018	2011	2018	Assuming 100% of changes in QALE are due to medical care	Assuming 80% of changes in QALE are due to medical care	Assuming 50% of changes in QALE are due to medical care
Q1 (lowest)	918	1448	40.3	41.0	62 567	78 209	125 134
Q2	839	1439	40.8	42.2	38 265	47 831	76 530
Q3	656	1236	41.3	42.8	37 524	46 905	75 049
Q4	749	1096	42.0	43.2	25 405	31 757	50 811
Q5 (highest)	654	1199	43.0	44.2	43 111	53 889	86 222

Sources: Authors’ analysis of data from the Korean Health Panel Study and the Korean National Health Insurance Service for the years 2011-2018. Note: The value of health care spending was assessed by dividing lifetime per capita health care spending by quality-adjusted life expectancy (QALE). This metric was estimated under three alternative assumptions regarding the extent to which improvements in QALE are attributable to medical care. First, we assumed that 100% of the observed gains in QALE were due to medical advancements. Second, given that mortality from external causes in Korea declined by approximately 20% between 2011 and 2018, an additional analysis was conducted assuming that the remaining 80% of health improvements were attributable to medical care. Third, drawing on prior studies suggesting that approximately half of the gains in life expectancy are attributable to medical interventions, we assumed that 50% of the observed improvements in QALE were the result of increased medical care utilization.

### Underlying mechanisms

**Figure 2.**
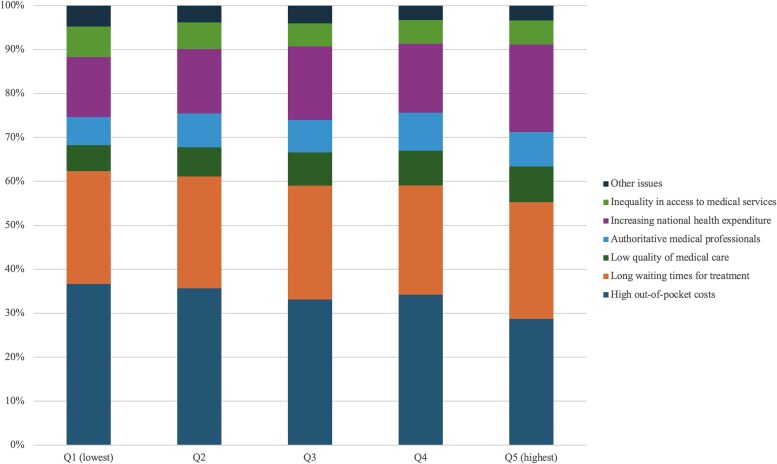
Self-reported perception of major issues in the Korean health care system among adults by household income in South Korea. Sources: Authors’ analysis of data from the Korean Health Panel Study for the years 2011-2018. Note: We assessed perceptions of the health care system based on each respondent's top concern, selected from seven mutually exclusive categories: high out-of-pocket costs, long wait times, low quality of care, authoritarian provider behavior (eg, lack of patient-provider communication or shared decision-making), rising national expenditures, inequity in access, and other issues.

We identified several potential mechanisms underlying income-based differences in the value of health care among adults in South Korea. The most frequently reported concerns about the health care system were high out-of-pocket costs, long waiting times, and rising national health expenditures (Figure 2). Of these, high out-of-pocket costs were cited most often, particularly by adults in the lowest-income quintile. This concern declined with increasing income, from 36.7% in the lowest quintile to 28.7% in the highest.

Adults in lower-income groups also reported worse health status, with disparities most pronounced in the lowest-income quintile (Table 2). The adjusted prevalence of major chronic conditions was higher in the lowest vs the highest-income quintile for cancer (adjusted difference: 0.2% points [95% CI: −0.2 to 0.6]), cerebrovascular disease (1.9 [1.6-2.3]), and heart disease (2.5 [2.0-2.9]). Functional limitations were significantly more prevalent in the lowest-income quintile, with differences of 6.7 (6.1-7.3) for disability, 5.5 (4.9-6.0) for limitations due to illness or injury, and 2.1 (1.7-2.5) for cognitive impairment. Clinical risk indicators followed similar patterns, with higher rates of severe depression (5.2 [4.2-6.1]), hypertension (2.1 [1.0-3.2]), diabetes (3.3 [2.3-4.3]), elevated C-reactive protein (3.9 [2.5-5.2]), and abnormal high-density lipoprotein (HDL) cholesterol (3.3 [2.1-4.5]).

**Table 2.** Potential underlying mechanisms for income-based disparities among adults in South Korea.

	Adjusted estimates, % (95% CI)					Differences (Q1 vs Q5), percentage points (95% CI)
	Q1 (lowest)	Q2	Q3	Q4	Q5 (highest)	
Health status						
Comorbidities						
Cancer	3 (2.7-3.3)	2.5 (2.2-2.7)	2.4 (2.2-2.7)	2.5 (2.2-2.7)	2.8 (2.5-3.1)	0.2 (−0.2 to 0.6)
Cerebrovascular diseases	3.2 (2.9-3.5)	2 (1.8-2.2)	1.7 (1.5-1.9)	1.6 (1.4-1.8)	1.3 (1.1-1.5)	1.9 (1.6-2.3)
Heart diseases	4.9 (4.5-5.3)	3.3 (3-3.5)	2.9 (2.6-3.1)	2.4 (2.2-2.6)	2.4 (2.2-2.7)	2.5 (2-2.9)
Functional status						
Disabled	9.1 (8.6-9.7)	4.9 (4.6-5.3)	3.6 (3.3-3.9)	2.5 (2.3-2.7)	2.4 (2.2-2.6)	6.7 (6.1-7.3)
Limitation due to diseases or injuries	7.4 (6.9-7.9)	3.3 (3-3.6)	2.7 (2.4-2.9)	2.2 (2-2.4)	1.9 (1.7-2.1)	5.5 (4.9-6)
Cognitive limitation	3.9 (3.5-4.2)	2.8 (2.5-3.1)	2 (1.8-2.3)	2 (1.8-2.3)	1.8 (1.6-2)	2.1 (1.7-2.5)
Clinical reports						
Severe depression (PHQ-9)	5.8 (4.9-6.7)	2.1 (1.6-2.6)	1.4 (1-1.9)	1.2 (0.8-1.6)	0.6 (0.3-0.8)	5.2 (4.2-6.1)
Hypertension (systolic and diastolic blood pressure)	20.7 (19.9-21.5)	20 (19.2-20.7)	19.7 (19-20.4)	18.6 (17.9-19.3)	18.6 (17.9-19.3)	2.1 (1-3.2)
Diabetes (HbA1C)	12.5 (11.7-13.3)	11.5 (10.8-12.1)	10.7 (10.1-11.3)	9.7 (9.1-10.2)	9.2 (8.7-9.8)	3.3 (2.3-4.3)
High C-reactive protein	11.2 (10.1-12.3)	8.5 (7.6-9.3)	8.3 (7.4-9.2)	7.1 (6.4-7.9)	7.3 (6.5-8.1)	3.9 (2.5-5.2)
Cholesterol						
High total cholesterol	32.6 (31.5-33.6)	37.4 (36.5-38.4)	38.8 (37.8-39.7)	39.1 (38.1-40)	39.4 (38.4-40.4)	−6.8 (−8.3 to −5.3)
Low HDL cholesterol	21 (20.1-21.9)	19.3 (18.6-20.1)	19.5 (18.7-20.3)	18.1 (17.3-18.8)	17.7 (17-18.4)	3.3 (2.1-4.5)
Risk factors						
Current smoker	22.3 (21.5-23.1)	20.9 (20.2-21.7)	19.8 (19.1-20.6)	18.1 (17.4-18.9)	15 (14.3-15.7)	7.3 (6.2-8.3)
Obese	6.6 (5.8-7.5)	6.7 (5.8-7.6)	5.7 (4.9-6.5)	4.4 (3.6-5.1)	4 (3.3-4.7)	2.7 (1.5-3.8)
Excessive drinking	13.9 (13-14.8)	14.1 (13.3-14.8)	14.1 (13.3-14.8)	14.3 (13.5-15)	13.1 (12.4-13.8)	0.8 (−0.4 to 2)
Unmet needs for health care						
Unmet needs for health care	26.9 (26-27.8)	23.6 (22.9-24.4)	20.9 (20.1-21.6)	19.4 (18.7-20.1)	17.3 (16.7-18)	9.6 (8.4-10.7)
Unmet needs due to financial reasons	16.6 (15.9-17.3)	11 (10.5-11.6)	8 (7.5-8.5)	5.9 (5.5-6.3)	3.3 (3.1-3.6)	13.3 (12.5-14.1)
Unmet needs due to non-financial reasons	11.1 (10.5-11.8)	11.1 (10.6-11.7)	11 (10.4-11.6)	10.3 (9.8-10.9)	10.1 (9.6-10.7)	1 (0.2-1.8)
Preventive care						
Medical checkup	50.6 (49.5-51.6)	60.2 (59.2-61.1)	65.5 (64.5-66.4)	70.1 (69.1-71)	74.4 (73.5-75.3)	−23.8 (−25.2 to −22.4)
Dental checkup	72.2 (70.9-73.6)	75.8 (74.7-77)	76.8 (75.7-78)	78.9 (77.9-80)	78.7 (77.7-79.7)	−6.5 (−8.2 to −4.7)
Breast cancer screening	52.7 (49.7-55.8)	57.2 (53.7-60.6)	61.5 (57.6-65.4)	58.9 (54.3-63.4)	62.9 (58.1-67.7)	−10.2 (−16.1 to −4.3)
Colorectal cancer screening	49.5 (47.1-51.9)	59.3 (56.7-61.8)	61 (58.1-63.8)	61.2 (57.9-64.5)	66.4 (63-69.8)	−16.9 (−21.2 to −12.5)
Cervical cancer screening	94.1 (93.7-94.6)	98.9 (98.7-99.1)	99 (98.8-99.2)	98.9 (98.7-99.1)	98.8 (98.6-98.9)	−4.6 (−5.1 to −4.1)

Sources: Authors’ analysis of data from the Korean Health Panel Study and the Korean National Health and Nutrition Examination Survey for the years. Note: We constructed binary indicators across four domains. The first domain—health status—included comorbidities (cancer, cerebrovascular disease, and heart disease), functional limitations (disability, illness-related limitations, and cognitive impairment), and clinical indicators (severe depression assessed by PHQ-9; hypertension via systolic/diastolic blood pressure; diabetes via HbA1c; elevated C-reactive protein; and abnormal cholesterol based on total and HDL levels). The second domain—behavioral risk factors—included current smoking, obesity, and excessive alcohol consumption. The third—unmet health care needs—captured both financial and non-financial barriers to care. The fourth—utilization of preventive services—included general medical and dental checkups as well as cancer screenings (breast, colorectal, and cervical. For these analyses, we conducted logistic regression after adjusting for age, sex, their interaction terms, and year and estimated demographic-adjusted differences by income.

Behavioral risk factors were also more prevalent in the lowest-income quintile, with smoking rates 7.3 (6.2-8.3) percentage points higher and obesity rates 2.7 (1.5-3.8) percentage points higher than in the highest-income quintile. However, differences in excessive alcohol consumption were minimal.

Unmet health care needs were highest in lower-income groups. In the lowest-income group, 26.9% reported unmet needs compared to 17.3% in the highest (9.6 [8.4-10.7]), largely driven by financial barriers (16.6% vs 3.3%; 13.3 [12.5-14.1]). Non-financial barriers varied little by income.

Preventive care utilization was consistently lower in lower-income groups. Compared to the highest-income quintile, the lowest-income quintile had lower rates of medical checkups (−23.8 [−25.2, −22.4]) and dental checkups (−6.5 [−8.2, −4.7]). Screening rates were also significantly lower for breast cancer (−10.2 [−16.1, −4.3]), colorectal cancer (−16.9 [−21.1, −12.5]), and cervical cancer (−4.6 [−5.1, −4.1]).

## Discussion

Previous research has assessed the value of health care spending in South Korea at the aggregate population level.^9^ Our study extends this work by examining how value varies across income groups, providing insights into the distribution of health gains relative to spending. Between 2011 and 2018, both per capita health care spending and QALE increased across all income quintiles; however, these increases were not even. Adults in the lowest-income quintile derived the least value from health care, while middle- and higher-income quintiles achieved greater value, though the highest value was not observed among those in the highest-income quintile.

While earlier research often examined disparities in access, utilization, or outcomes separately,^32-35^ our analysis provides a more integrated assessment by jointly examining health care costs and health gains. We found that per capita spending was similar across income groups, but the lowest-income quintile experienced much smaller gains in QALE. This contrasts with the common expectation that additional health care spending yields greater benefits for lower-income populations, reflecting the principle of diminishing returns. Two factors likely account for this observation. First, earlier studies did not adjust for key differences such as age and sex, which can confound results; our analysis accounted for these factors to better isolate the effects of spending. Second, South Korea's more generous cost-sharing protections for lower-income groups may encourage higher use of health care, including low-value services. Thus, additional spending does not necessarily translate into proportionally greater health gains.

While we did not identify causal pathways directly, secondary data provide several plausible explanations. Adults in the lowest-income quintile had poorer baseline health, including comorbidities, functional limitations, and clinical risk factors, making health gains more difficult to achieve. Additionally, despite greater health needs, these adults faced persistent barriers to accessing care, particularly financial constraints. These barriers were reflected in lower utilization of preventive services,^36^ potentially limiting the returns on health care investment.

Notably, the highest value was not observed among adults in the highest-income group, indicating a non-linear relationship between income and the value of health care. The highest value was in the second highest-income quintile, suggesting diminishing returns to health care spending at higher income levels and highlighting potential inefficiencies in the system. One explanation is that higher-income groups may consume more low-value or marginally beneficial services. This pattern may partly stem from unintended consequences of South Korea's health insurance expansion over the past two decades, which have improved access to care,^37-39^ but have not explicitly addressed the value or appropriateness of services. Consequently, broader coverage may have inadvertently encouraged inefficient utilization.^40,41^

These findings underscore the need for health policy in South Korea to prioritize both equity and value.^42^ To improve efficiency and fairness, targeted strategies are essential. For lower-income populations, expanding financial protection and improving access specifically to high-value preventive and primary care services—rather than broadly lowering costs for all care—should be key policy priorities. However, health systems alone cannot eliminate disparities rooted in broader social determinants.^43,44^ Meaningful progress will require coordinated efforts to address upstream factors like income inequality. Simultaneously, reducing the overuse of low-value services can enhance system efficiency. Strengthening value-based care models and implementing evidence-based clinical appropriateness guidelines can help ensure that health care spending yields meaningful health improvements across the entire population.

Although this study focuses on South Korea, the findings have broader implications for understanding how income inequality interacts with different health system designs. In countries with greater income inequality and more fragmented health systems, such as the United States, disparities in the value of health care spending may be even more pronounced. Conversely, in countries with more equity-oriented systems, these differences might be less evident. Future comparative studies across diverse health systems could help clarify how income inequality and system design together shape the relationship between health care spending and health outcomes.

## Conclusions

This study highlights persistent income-based disparities in the value of health care in South Korea. Adults in the lowest-income quintile derived the least value, largely due to poorer baseline health and limited access to care. These findings point to structural inequities within the South Korean health system and emphasize the need for targeted policy interventions that promote more equitable health care value.

## Supplementary Material

qxaf145_Supplementary_Data
