# Supplementary material for: Income-related disparities in the value of health care in South Korea
Source: Health Aff Sch. 2025 Jul 22;3(8):qxaf145. doi: 10.1093/haschl/qxaf145 (PMC12359133; doi:10.1093/haschl/qxaf145)
Supplement: qxaf145_Supplementary_Data [file qxaf145_supplementary_data.zip › HAS_R1_Appendix_Value of care by income in Korea.docx]

**Estimation of Visual Analog Scale (VAS) Scores**

We used three measures to assess health-related quality of life (HRQoL): VAS scores, EQ-5D scores, and self-reported good health. Among these, VAS scores were primarily employed to estimate quality-adjusted life expectancy (QALE). To validate this approach, we confirmed that the other two HRQoL measures showed similar temporal trends. However, because the Korean Health Panel Study collected VAS data only in 2011 and 2013, we adopted the methodology of Cutler et al. and developed a linear regression model to predict VAS scores for the years 2011 and 2018. As shown in the table, the model included age group, sex, disability status, and functional status as key covariates. Using this model, we then predicted individual-level VAS scores for each year.

We found that VAS scores were lower among individuals of older age, those with disabilities, and those with more severe functional limitations. While some associations did not reach statistical significance, most were statistically significant and in the expected direction.

| **Variables** | **Estimates (95% CI)** | **P value** |
| --- | --- | --- |
| Age group (REF: 18-24) |  |  |
| 25-34 | -1.6 (-2.4, -0.7) | 0 |
| 35-55 | -1.8 (-2.5, -1) | 0 |
| 45-54 | -2 (-2.8, -1.2) | 0 |
| 55-64 | -2.4 (-3.2, -1.6) | 0 |
| 65-74 | -3.1 (-4, -2.3) | 0 |
| 75+ | -3.7 (-4.7, -2.6) | 0 |
| Female (REF: Male) | -1.6 (-1.9, -1.2) | 0 |
| Disability (REF: none) |  |  |
| Moderate | -2.5 (-3.4, -1.5) | 0 |
| Severe | -1.3 (-2.7, 0.2) | 0.088 |
| Limited athletic ability (REF: none) |  |  |
| Moderate | -3 (-3.7, -2.3) | 0 |
| Severe | -3.6 (-8.3, 1.2) | 0.143 |
| Limited self-management (REF: none) |  |  |
| Moderate | 0.4 (-0.8, 1.6) | 0.55 |
| Severe | -2.4 (-6.8, 2) | 0.285 |
| Limited daily activities (REF: none) |  |  |
| Moderate | -2.2 (-3.1, -1.2) | 0 |
| Severe | -6.7 (-10.8, -2.5) | 0.002 |
| Pain (REF: none) |  |  |
| Moderate | -5.5 (-5.9, -5.1) | 0 |
| Severe | -15 (-16.6, -13.5) | 0 |
| Depressive symptoms (REF: none) |  |  |
| Moderate | -6.6 (-7.1, -6) | 0 |
| Severe | -16.5 (-19.3, -13.7) | 0 |
| Difficulty in seeing (REF: none) |  |  |
| Moderate | -1.1 (-1.5, -0.7) | 0 |
| Severe | -2.5 (-3.7, -1.3) | 0 |
| Difficulty in hearing (REF: none) |  |  |
| Moderate | -1.7 (-2.3, -1) | 0 |
| Severe | -1.1 (-2.4, 0.3) | 0.139 |
| Difficulty in eating (REF: none) |  |  |
| Moderate | -0.8 (-1.2, -0.4) | 0 |
| Severe | -2.2 (-2.7, -1.8) | 0 |
| Difficulty in memory (REF: none) | -1.7 (-2.5, -0.8) | 0 |
| Difficulty in decision-making (REF: none) | -3.4 (-5, -1.8) | 0 |
| Limitation due to illness (REF: none) | -2.7 (-3.7, -1.7) | 0 |
| Constant | 79.9 (79.2, 80.6) | 0 |

**Estimation of Quality-Adjusted Life Expectancy (QALE)**

To estimate QALE, we multiplied life expectancy at age 25 for a given age-sex-income group by estimated VAS scores for that age-sex-income group, producing a summary measure that captures both longevity and quality of life. Life expectancy estimates for males and females in each income group were estimated from beneficiary enrollment data from the National Health Insurance Service (NHIS), while VAS scores were obtained from the Korean Health Panel Study. Because these datasets could not be directly linked at the individual level, we calculated the average VAS scores by income group and year. QALE was then estimated by multiplying the sex-specific life expectancy at age 25 by the corresponding average VAS score for each income and year group. Although VAS scores vary by age, the results from the previous table indicate that the differences were relatively small beyond age 25, when controlling for other factors such as functional limitations. For instance, the average difference in VAS scores between individuals aged 25–34 and those aged 75 and older was approximately 2.1 points. Therefore, we did not fully adjust for age in our analysis, as such differences are unlikely to meaningfully affect our findings. We mention the assumption of the same income group across the life course in our discussion of limitations, and plan to undertake future analyses exploring QALE and the net value of healthcare in Korea in more detail, building upon these analyses.

**Comparison with Park et al. (JAMA Health Forum)**

This study differs from the work by Park et al. in several key respects. First, as highlighted by the title’s focus on “income-based disparities,” this analysis explicitly examines differences in value by income group, whereas Park et al. evaluated overall value without directly assessing income-based variation. Second, although the prior study reported spending levels by income quintile, it did not investigate differences in health gains or outcomes across income groups and therefore did not assess disparities in value, which is the central focus of this study. Third, the present study explores specific conditions by income group that may explain observed disparities, as shown in Table 2, whereas the earlier study did not analyze health gains or spending by individual condition. Fourth, the methodological approaches differ substantially. Park et al. used DALYs from the Global Burden of Disease to examine changes in health for Korea’s overall population. By contrast, this study estimates quality-adjusted life expectancy (QALE) using Korean Health Panel data along with income-specific life expectancy at age 25, and calculates value based on lifetime remaining spending, rather than on changes in average cross-sectional spending across baseline and follow-up years. Collectively, these distinctions demonstrate that while both studies address related topics, they do so with fundamentally different research objectives, populations, outcomes, and analytic methods.

**Appendix Table A. Baseline characteristics of adults (aged 18 years and older) by household income in South Korea.**

|  | Weighted % |  |  |  |  |
| --- | --- | --- | --- | --- | --- |
| Characteristics in 2011 | Q1 (lowest) | Q2 | Q3 | Q4 | Q5 (highest) |
| N | 2254 | 2253 | 2283 | 2231 | 2245 |
| Annual household income ($), mean (SD) | 17835 (3792) | 30443 (3379) | 42839 (3694) | 57655 (5028) | 93185 (26785) |
| Age, % |  |  |  |  |  |
| 18-25 | 8.3 | 10.1 | 12.8 | 15.0 | 17.5 |
| 26-44 | 22.4 | 44.4 | 46.3 | 46.5 | 39.7 |
| 45-64 | 36.1 | 33.4 | 34.9 | 33.3 | 39.4 |
| 65+ | 33.2 | 12.1 | 5.9 | 5.2 | 3.4 |
| Female, % | 54.8 | 49.7 | 50.5 | 50.2 | 51.9 |
| Disability, % |  |  |  |  |  |
| Moderate | 6.9 | 3.6 | 1.8 | 2.1 | 1.3 |
| Severe | 4.5 | 1.6 | 1.0 | 0.6 | 0.7 |
| Married, % | 62.1 | 70.0 | 69.6 | 69.6 | 67.1 |
| Employed, % | 51.4 | 61.0 | 64.3 | 65.7 | 64.2 |
| Health insurance, % |  |  |  |  |  |
| National health insurance | 89.5 | 97.5 | 99.5 | 99.5 | 100 |
| Medical aid | 10.5 | 2.5 | 0.5 | 0.5 | 0.0 |
| Comorbidities, % |  |  |  |  |  |
| Arthritis | 18.1 | 7.3 | 4.4 | 4.7 | 3.8 |
| Cancer | 3.3 | 2.2 | 1.3 | 1.7 | 2.3 |
| Cerebrovascular | 3.5 | 1.5 | 1.0 | 1.1 | 0.5 |
| Diabetes | 10.9 | 6.0 | 4.2 | 3.7 | 4.0 |
| Heart disease | 5.9 | 2.7 | 1.8 | 1.7 | 1.2 |
| Hypertension | 28.4 | 14.9 | 10.4 | 11.6 | 9.6 |
| Hyperlipidemia | 9.9 | 6.1 | 4.3 | 4.7 | 4.5 |
| Depression | 5.3 | 2.5 | 1.7 | 1.8 | 1.3 |

Data source: Korea Health Panel Study for 2011-2018.

To address substantial differences in sample characteristics, individuals in the bottom 10% of annual household income were excluded from the analysis. Subsequently, annual household income was divided into quintiles, and survey weights were applied to ensure the sample characteristics were representative of the South Korean population. Disease diagnoses were based on administrative claims data, which may underestimate the true prevalence of certain conditions. This limitation highlights the need to integrate supplementary data sources to enhance diagnostic accuracy and provide a more comprehensive assessment of population health.

**Appendix Figure A. Distribution of household income in South Korea.**


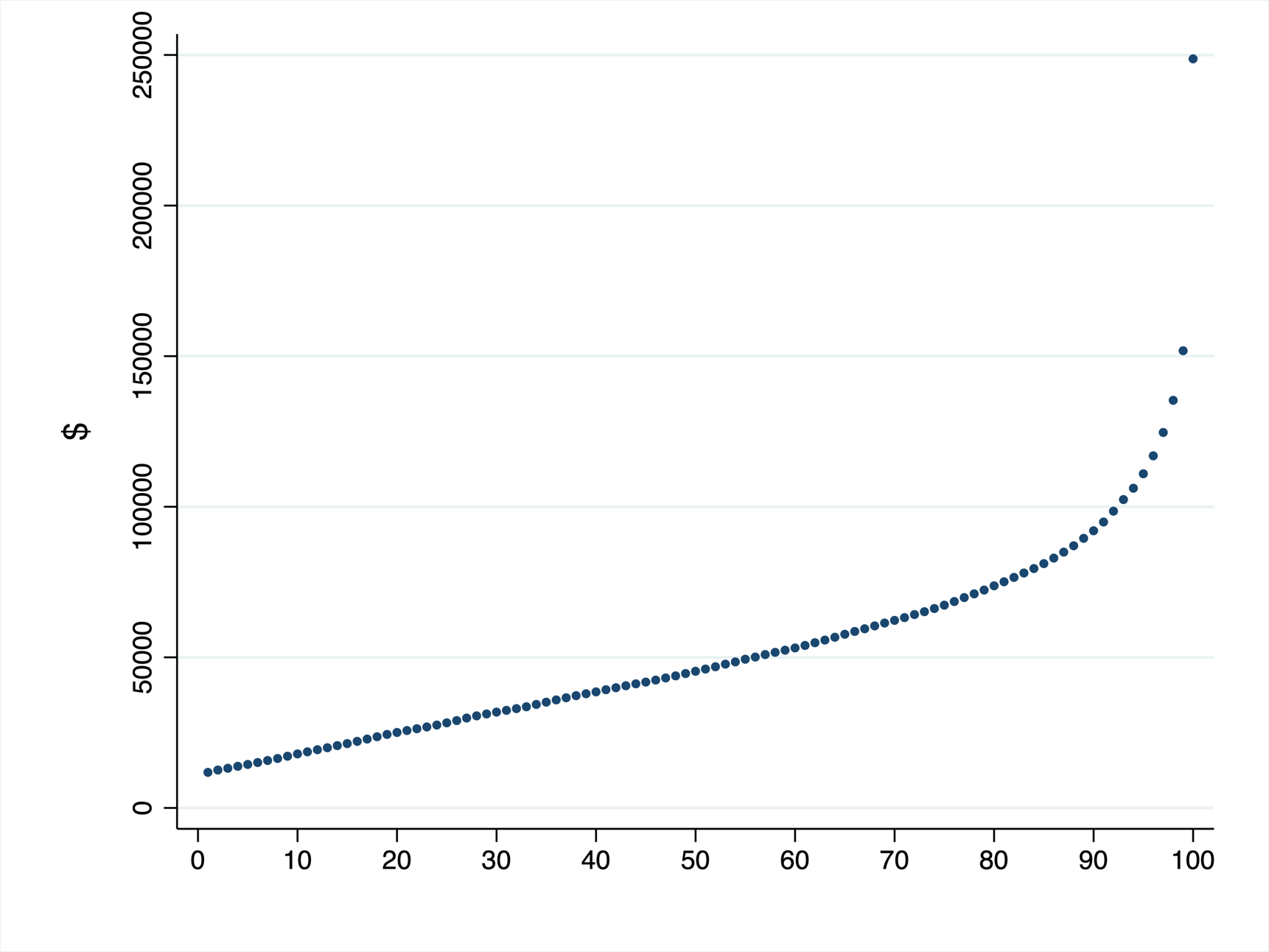


**Appendix Figure B. Trends in average annual household income by household income in South Korea.**


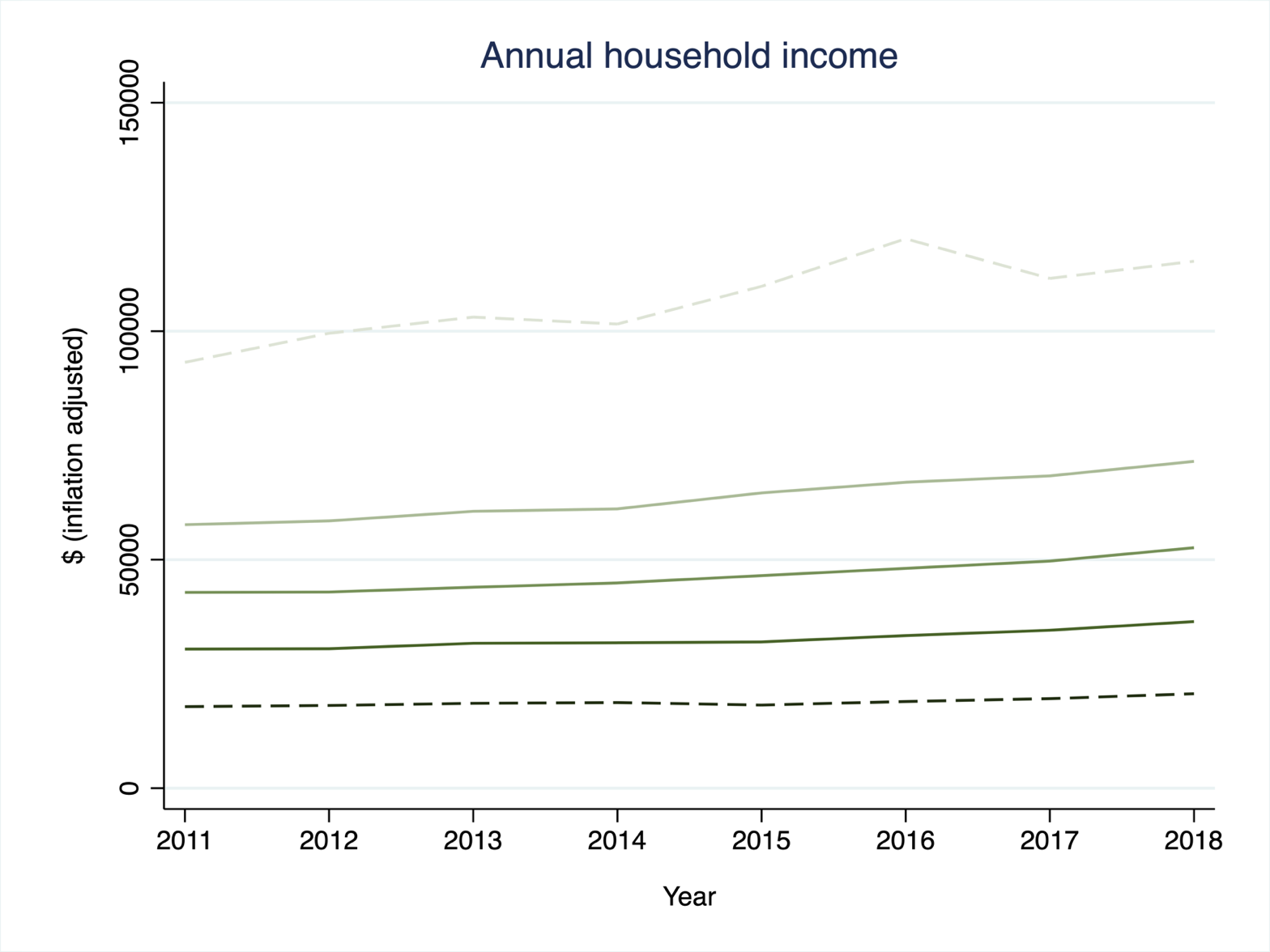


**
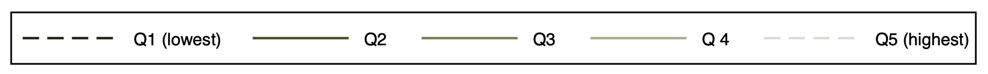
**

**Appendix Figure C. Trends in health status among adults by household income in South Korea.**


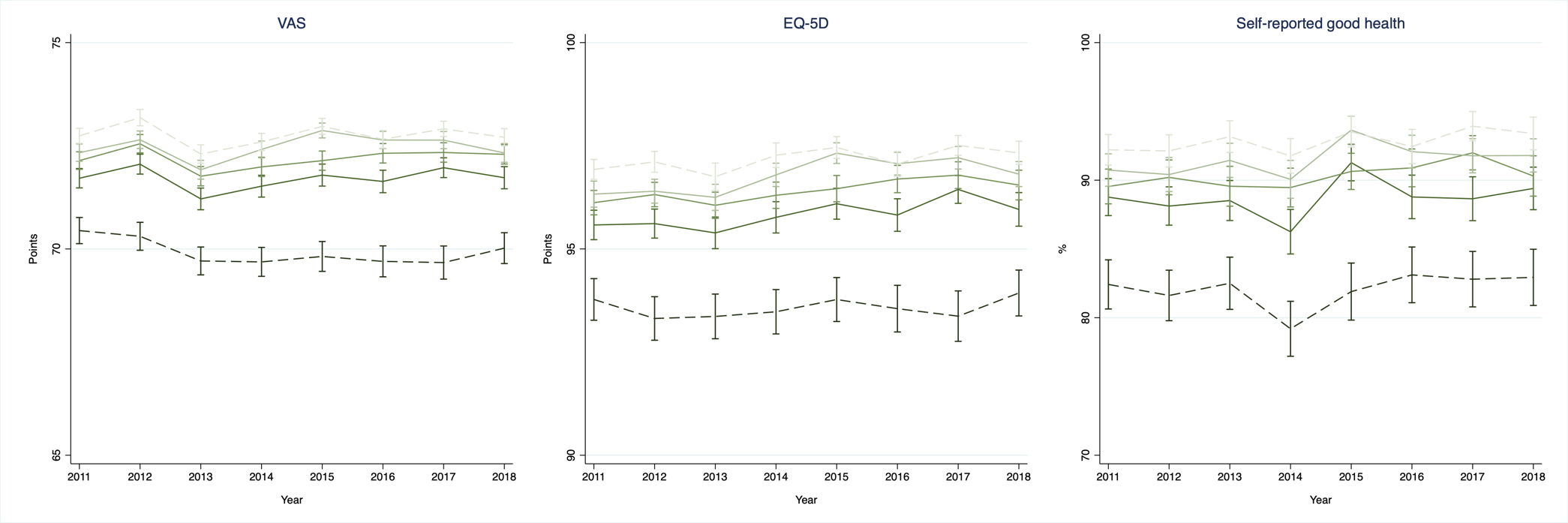


**
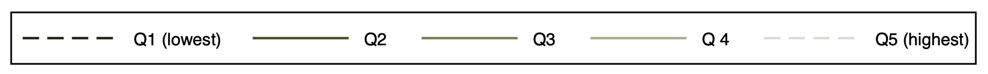
**

**Appendix Figure D. Trends in health outcomes among adults by household income in South Korea.**

**
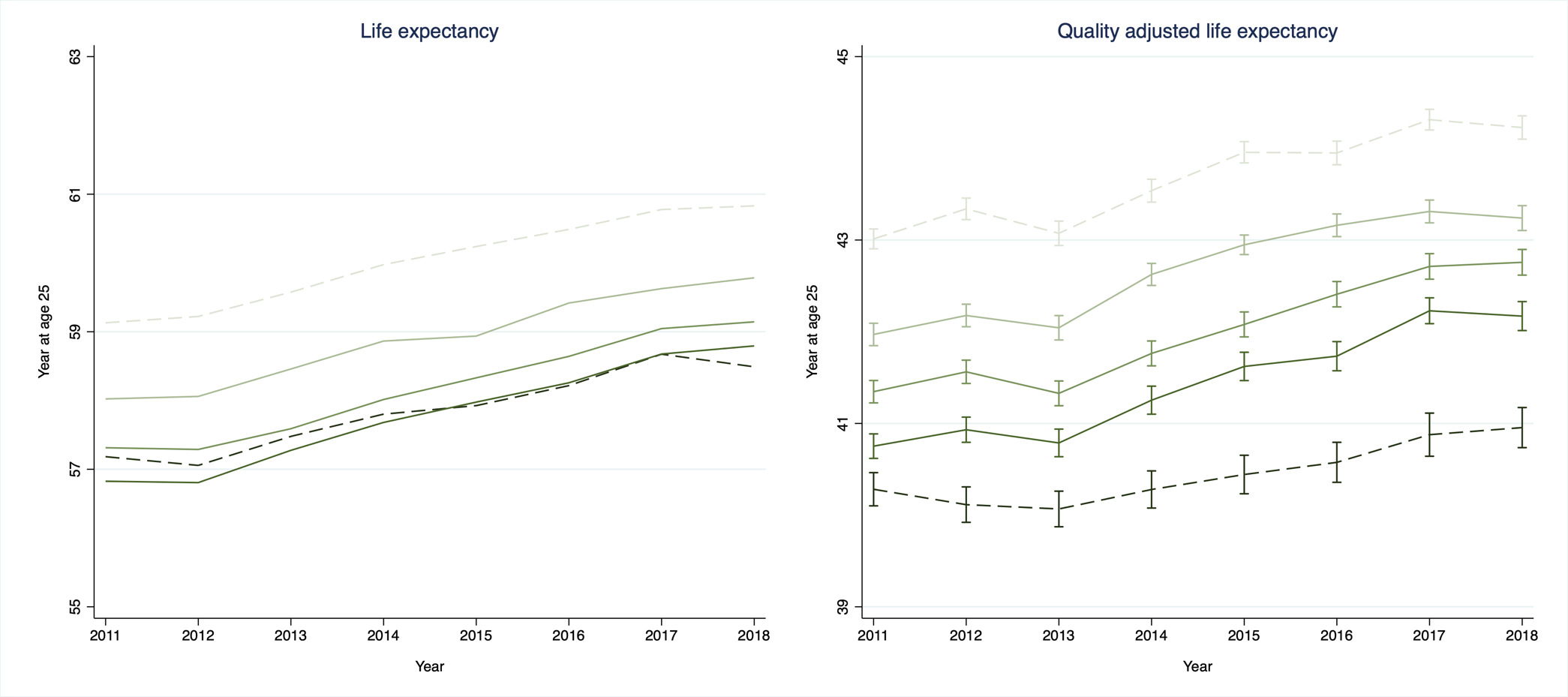
**

**
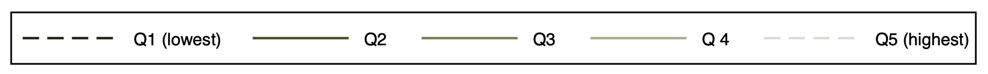
**
